# Chemical Composition, Antioxidant Potential, and Acetylcholinesterase Inhibitory Activity of the Essential Oil from *Croton alnifolius* Lam.

**DOI:** 10.3390/molecules31010061

**Published:** 2025-12-24

**Authors:** Claudia Cruz, Pablo Muñoz, Nixon Cumbicus, Vladimir Morocho, Omar Malagón

**Affiliations:** 1 Departamento de Química, Universidad Técnica Particular de Loja (UTPL), Loja 1101608, Ecuador; omalagon@utpl.edu.ec; 2 Carrera de Bioquímica y Farmacia, Universidad Técnica Particular de Loja (UTPL), Loja 1101608, Ecuador; pamunoz11@utpl.edu.ec; 3 Departamento de Ciencias Biológicas y Agropecuarias, Universidad Técnica Particular de Loja (UTPL), Calle Marcelino Champagnat s/n, Loja 110107, Ecuador; nlcumbicus@utpl.edu.ec; 4 Independent Researcher, Loja 110102, Ecuador; vladimirmorocho@gmail.com

**Keywords:** *Croton alnifolius*, essential oil, antioxidant, cholinesterase

## Abstract

This study reports the first chemical characterization of the essential oil of *Croton alnifolius*. A very low yield of 0.028% ± 0.0012 (*w*/*w*) was obtained by steam distillation for 4 h using a Clevenger-type apparatus. The chemical composition of the oil was analyzed by gas chromatography coupled with mass spectrometry (GC–MS) for compound identification and by gas chromatography with a flame ionization detector (GC–FID) for quantification. A total of 49 compounds were identified, representing 94.65% of the total oil composition. The chemical profile was dominated by hydrocarbon sesquiterpenes (53.11%) and hydrocarbon monoterpenes (32.20%). The major constituents included (*E*)-caryophyllene (17.42%), α-pinene (14.53%), myrcene (9.51%), germacrene D (9.92%), and β-chamigrene (5.48%). The biological activity of the essential oil was also evaluated: it exhibited weak antimicrobial activity against *Enterococcus faecium* with a Minimum Inhibitory Concentration (MIC) value of 1000 μg/mL, strong antioxidant potential in the ABTS assay (SC_50_ = 28.43 ± 1.0 μg/mL), and moderate acetylcholinesterase inhibitory activity (61.74 ± 1.02 μg/mL). These results indicate that the unique sesquiterpene rich chemical profile of *C. alnifolius* contributes to its antioxidant and neuroprotective potential, supporting its relevance as a promising source of bioactive natural products.

## 1. Introduction

Medicinal and aromatic plants, especially those with ethnopharmacological uses, have been utilized as natural sources of remedies and healthcare for millennia, forming the foundation of many traditional medical systems worldwide [1,2]. Consequently, essential oils are highly valued for their biological properties and diverse industrial applications. In cosmetics, they are used for their aromatic qualities and skin benefits, while in the food industry they serve as natural flavoring agents and preservatives due to their antimicrobial and antioxidant effects. Moreover, their therapeutic potential, including antimicrobial, anti-inflammatory, and anticancer activities, supports their pharmacological relevance [3,4].

Euphorbiaceae is one of the largest families of angiosperms with global distribution, and several species produce essential oils with diverse chemical compositions and biological activities [5]. The genus *Croton* (Euphorbiaceae) is one of the most diverse within the family, comprising approximately 1200–1300 species distributed across tropical and subtropical regions of the world. Its species are known for producing a wide variety of secondary metabolites, including flavonoids, lignins, coumarins, tannins, alkaloids, cyanogenic glycosides, and glucosinolates. In particular, their volatile constituents, mainly essential oils, exhibit a broad spectrum of biological activities such as antioxidant, antimicrobial, anti-inflammatory, cytotoxic, antitumor, insecticidal, antiparasitic, anti-ulcerogenic, antinociceptive, myorelaxant, antispasmodic, anxiolytic, anthelmintic, and vasorelaxant effects [6,7,8]. This remarkable chemical and biological diversity has made *Croton* species valuable targets for phytochemical and pharmacological investigations, contributing to the discovery of new bioactive natural products with therapeutic potential.

Moreover, essential oils, including those from *Croton* species, demonstrate significant antioxidant potential by neutralizing free radicals and mitigating oxidative stress and cellular damage. Consequently, this activity contributes to slowing skin aging and reducing inflammation by decreasing proinflammatory cytokine expression. Therefore, essential oils are considered promising agents for preventing and managing chronic diseases, including diabetes, hyperlipidemia, and hypertension [9,10,11].

Another important biological property is acetylcholinesterase inhibition, which increases acetylcholine levels. Since reduced acetylcholine is associated with neurodegenerative diseases, inhibiting this enzyme may help slow disease progression [12]. Essential oils and their chemical constituents act on the central nervous system by inhibiting acetylcholinesterase [13]. Due to their small molecular size and lipophilicity, essential oil components can cross the blood–brain barrier. Moreover, their volatility facilitates inhalation administration, avoiding metabolic degradation and preserving active compounds [14].

Therefore, this study focuses on obtaining and characterizing the essential oil from *Croton alnifolius* Lam., evaluating its antimicrobial activity, and highlighting its notable antioxidant and acetylcholinesterase inhibitory properties. Additionally, a literature review on *Croton* essential oils was conducted to emphasize their relevance in oxidative stress reduction and neuroprotection as natural sources for therapeutic applications.

*C. alnifolius* is native to the region spanning from Ecuador to Peru and grows primarily in the wet tropical biome. It is a shrub species ranging from 80 cm to 1.5 m in height, characterized by simple, alternate leaves with stipules and an elliptic to ovate lamina with entire or slightly dentate margins. The small unisexual flowers are arranged in racemose inflorescences at the upper part of the plant and display a yellow coloration [15,16].

Considering the growing interest in natural products with antioxidant and neuroprotective properties, the study of *C. alnifolius* essential oil represents a valuable opportunity to expand current knowledge of the genus. Understanding its overall chemical composition and associated biological properties can contribute to recognizing species with potential pharmacological applications. Therefore, this study aimed to characterize the volatile chemical profile of the essential oil from *C. alnifolius* and to evaluate its antioxidant, antimicrobial, and acetylcholinesterase inhibitory activities, providing a scientific basis for its potential use in the development of natural antioxidant and neuroprotective agents.

## 2. Results

### 2.1. Yield and Chemical Composition

The essential oil of *C. alnifolius* was obtained by steam distillation for 4 h using a Clevenger-type apparatus. The yield was calculated as the ratio between the weight of the essential oil obtained and the dry weight of the plant material (*w*/*w*%), resulting in a very low yield of 0.028% ± 0.0012. Furthermore, a total of 49 compounds were identified by gas chromatography–mass spectrometry (GC–MS) and confirmed by gas chromatography with flame ionization detection (GC–FID), representing 94.65% of the total essential oil content. Of the total composition, 32.20% corresponded to hydrocarbon monoterpenes, 1.59% to oxygenated monoterpenes, 53.11% to hydrocarbon sesquiterpenes, 5.00% to oxygenated sesquiterpenes, and the remaining 2.76% to other compounds. The major constituents were (*E*)-caryophyllene (17.42%), α-pinene (14.53%), myrcene (9.51%), germacrene D (9.92%), and β-chamigrene (5.48%). The rest of the identified compounds were present at concentrations below 3% and were therefore considered minor constituents. The detailed chemical composition is presented in Table 1, where the compounds are presented in order of elution on the TR5-MS capillary column. For each constituent, the experimental retention index, the corresponding literature retention index, and the mean relative percentage are provided.

**Figure 1:**
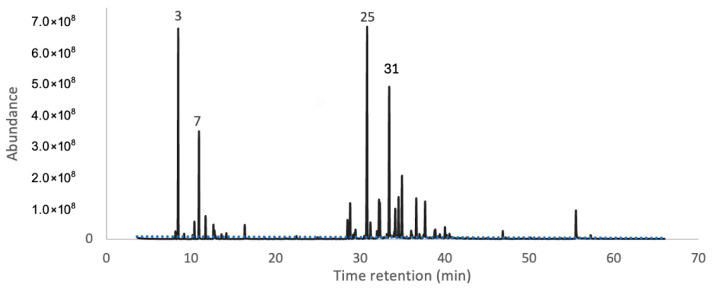
GC-MS chromatogram of essential oil obtained with a TR5-MS capillary column. The numbers correspond to the main components.

In Figure 1, the GC–MS chromatogram of the essential oil of *C. alnifolius* is presented, illustrating the complexity and diversity of volatile constituents in the sample. Each peak in the chromatogram corresponds to an individual compound, identified based on its retention time and mass spectral data. The integration and comparison of these peaks enabled a more precise characterization of the essential oil chemical profile.

### 2.2. Antimicrobial Activity of Essential Oil

The antimicrobial activity of the essential oil of *C. alnifolius* was evaluated against a panel of bacterial and fungal strains, and the results of the antimicrobial activity are presented in Table 2. The oil exhibited weak antibacterial activity against *Enterococcus faecium* (ATCC^®^ 27270) with an MIC value of 1000 μg/mL and antifungal activity against *Aspergillus niger* (ATCC^®^ 6275) with 4000 μg/mL. The other tested strains showed no growth inhibition at the concentrations evaluated, whereas the assays were validated using ampicillin (1 g/mL) and ciprofloxacin (1 mg/mL) as positive controls for bacteria, and amphotericin B (250 μg/mL) for fungi.

### 2.3. Antioxidant Activity

The antioxidant activity of *C. alnifolius* essential oil was evaluated using the DPPH (2,2-diphenyl-1-picrylhydrazyl) and ABTS (2,2-azino-bis(3-ethylbenzothiazoline-6-sulfonic acid)) radicals, with Trolox employed as the positive control (Table 3). Results are expressed as SC_50_ (µg/mL), representing the concentration required to reduce 50% of the free radical, along with the corresponding standard deviation. In addition, for the ABTS assay, the Trolox equivalent antioxidant capacity (TEAC) was calculated. In the DPPH assay, the essential oil exhibited an SC_50_ value of 1903.29 ± 1.26 µg/mL, indicating a very low antioxidant activity compared with Trolox. In contrast, in the ABTS assay, the essential oil showed an SC_50_ value of 28.43 ± 1.0 µg/mL, demonstrating a remarkably higher antioxidant activity, even exceeding that of Trolox.

### 2.4. Acetylcholinesterase Inhibitory Activity

**Figure 2:**
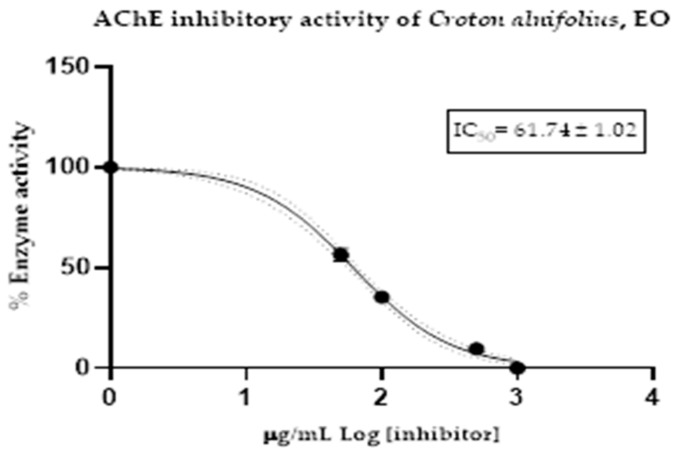
Half-maximum inhibitory concentration of *C. alnifolius* essential oil against acethylcholinesterase.

The acetylcholinesterase (AChE) inhibitory activity of *C. alnifolius* essential oil was evaluated using Ellman’s method [19]. Results are expressed as the half-maximal inhibitory concentration (IC_50_, µg/mL–nM) with the corresponding standard deviation. Donepezil was used as the positive control. The essential oil of *C. alnifolius* exhibited an IC_50_ value of 61.74 ± 1.02 µg/mL against AChE, whereas donepezil showed an IC_50_ of 12.40 ± 1.35 µg/mL. The dose–response inhibition curve is shown in Figure 2, illustrating that increasing concentrations of the essential oil led to a progressive decrease in AChE activity. The IC_50_ value was determined based on this concentration–response curve.

## 3. Discussion

The essential oil of *Croton alnifolius* was obtained through steam distillation over a period of 4 h, resulting in a yield of 0.0289%. According to the criteria established by the Science and Technology for Development Program (CYTED) [20], this yield is considered low, as yields above 10 mL/kg are classified as high, those between 5 and 10 mL/kg as intermediate, and values below 5 mL/kg as low. Investigations on other species of the *Croton* genus show generally low essential oil yields. For example, *C. adipatus*, *C. thurifer*, and *C. collinus* exhibited yields of 0.01%, 0.07%, and 0.001%, respectively [21]. Furthermore, *C. ferrugines* presented a yield similar to that observed for *C. alnifolius*, with a value of 0.02% [22]. In contrast, *C. greveanus*, *C. geayi*, and *C. borarium* exhibited higher yields of 0.96%, 0.72%, and 0.68%, while *C. zambesicus* reached 0.29% [23,24]

Essential oil yield is influenced by multiple biological and technical factors. Climatic conditions play a significant role; for example, yields tend to be higher during rainy seasons compared to dry periods [25]. Distillation parameters, particularly the applied pressure, also significantly affect both yield and the physicochemical properties of the oil [26]. Moreover, geographic origin is another important determinant: higher altitudes are associated with increased essential oil yields, likely due to environmental conditions such as lower temperatures, reduced atmospheric pressure, and greater exposure to ultraviolet radiation, which stimulate the production of secondary metabolites [27].

Gas chromatography–mass spectrometry (GC–MS) analysis of the essential oil of *C. alnifolius* allowed the identification of 49 compounds, accounting for 94.65% of the total oil composition. Revealing a substantial presence of hydrocarbonated monoterpenes represented 32.20%, and hydrocarbonated sesquiterpenes 53.11%, the other groups with values less than 5%. The major constituents included (*E*)-Caryophyllene (17.42%), α-Pinene (14.53%), Myrcene (9.51%), Germacrene D (9.92%), and β-Chamigrene (5.48%), while other components were present at concentrations below 3% and thus considered minor constituents. This study constitutes the first report of the essential oil composition of *C. alnifolius*. Comparative analysis with other *Croton* species revealed both similarities and species-specific variations. For instance, the essential oil of *C. ferrugineus* contains 43.5% sesquiterpenes and 34.98% monoterpenes, with major constituents including (*E*)-Caryophyllene (20.47%), Myrcene (11.47%), β-Phellandrene (10.55%), Germacrene D (7.60%), Linalool (7.34%), and Humulene (5.49%) [22]. Similarly, *C. rivulifolius* presents a predominance of sesquiterpenes (68.21%) and low monoterpene content (1.21%), with γ-Muurolene (15.3%), (*E*)-Caryophyllene (11.7%), β-Elemene (6.4%), and α-Humulene (5.7%) as major constituents [28]. In *C. hirtus*, sesquiterpenes account for 95.4% of the oil, dominated by β-Caryophyllene (32.8%), Germacrene D (11.6%), β-Elemene (9.1%), α-Humulene (8.5%), and Caryophyllene oxide (5.0%) [6]. These comparisons indicate that the essential oil composition of *C. alnifolius* exhibits a strong chemical affinity with other species within the genus, particularly due to the substantial sesquiterpene content. Moreover, the recurrent presence of α-Pinene, Myrcene, and Germacrene D across species suggests a conserved chemical signature characteristic of the *Croton* genus. This pattern may reflect shared biosynthetic pathways and ecological adaptations that influence secondary metabolite production.

The predominance of sesquiterpenes in *C. alnifolius* and other *Croton* species is noteworthy given their recognized biological activities, including anti-inflammatory, antimicrobial, and antioxidant effects. The presence of compounds such as (*E*)-Caryophyllene, α-Pinene, and Myrcene further highlights the potential pharmacological relevance of *C. alnifolius*, warranting further studies on its bioactive properties and possible applications in pharmaceutical and industrial contexts.

Overall, the GC–MS profile presented here not only expands the phytochemical knowledge of the genus but also provides a chemical basis for future comparative and functional studies, contributing to the understanding of the chemical diversity and ecological significance of *Croton* essential oils.

The essential oil of *C. alnifolius* exhibited selective antimicrobial activity. The minimum inhibitory concentration (MIC) was determined to be 1000 μg/mL against *Enterococcus faecium* and 4000 μg/mL against *Aspergillus niger*, while no inhibition was observed against the remaining tested strains up to the maximum tested concentration (1000 μg/mL for bacteria and 4000 μg/mL for fungi). According to the MIC classification proposed by Freires et al. (2015) [29], values between 501 and 1000 μg/mL indicate moderate activity, whereas MIC values above 2001 μg/mL are considered inactive. Therefore, the essential oil of *C. alnifolius* can be regarded as moderately active against *E. faecium* and inactive against *A. niger*.

Although α-pinene and β-caryophyllene, two major components of this oil have been widely reported to exhibit antibacterial and antifungal properties [30], the overall antimicrobial response of *C. alnifolius* was relatively weak. This limited effect may arise from the low concentration of individual active constituents or from the occurrence of antagonistic interactions within the complex mixture of volatile compounds, which can suppress the activity of otherwise bioactive molecules [31]. Furthermore, the extraction method can also influence the biological performance of the oil. Conventional techniques such as hydrodistillation or steam distillation are often inefficient and may cause thermal degradation, leading to the loss of volatile compounds and a consequent reduction in antimicrobial efficacy [32]. Comparative studies within the *Croton* genus support this interpretation: *C. hirtus* and *C. collinus*, both rich in β-caryophyllene (>35%), exhibited stronger inhibitory effects [6,21], whereas *C. ferrugineus*, with a composition similar to *C. alnifolius*, showed negligible antimicrobial activity [22]. These observations reinforce that antimicrobial efficacy depends not only on the abundance of specific terpenes but also on the overall chemical matrix, synergistic interactions, and possible volatility losses during testing [33].

Although MBC (Minimum Bactericidal Concentration) and MFC (Minimum Fungicidal Concentration) values were not determined in this study, future assays should include these parameters to better differentiate between bacteriostatic and bactericidal or fungistatic and fungicidal effects, there by strengthening the antimicrobial evaluation of *C. alnifolius* essential oil.

In relation to the antioxidant potential, the essential oil of *C. alnifolius* exhibited markedly different responses depending on the assay employed. In the DPPH method, the oil presented an SC_50_ value of 1903.29 ± 1.26 μg/mL, indicating very low antioxidant activity when compared with the positive control (Trolox). In contrast, the ABTS assay revealed an SC_50_ of 28.43 ± 1.0 μg/mL, representing an antioxidant activity that surpassed that of Trolox.

According to the classification proposed by Kusmardiyani et al. (2016) [34], essential oils with IC_50_ values below 50 μg/mL are considered to have very strong antioxidant activity, those between 50 and 100 μg/mL strong activity, those between 101 and 150 μg/mL moderate activity, and those above 150 μg/mL weak activity. Based on this classification, the essential oil of *C. alnifolius* demonstrated very strong antioxidant activity in the ABTS assay but only weak activity in the DPPH assay. This discrepancy between assays can be attributed to methodological differences. The ABTS method is recognized as more sensitive, likely due to its faster reaction kinetics; ABTS radicals rapidly reach a steady state with antioxidants, whereas DPPH requires several hours to stabilize [35]. Similarly, Floegel et al. (2011) [36] emphasized that ABTS provides a more precise and comprehensive evaluation of antioxidant potential compared to DPPH.

Previous studies have linked the antioxidant capacity of essential oils to the presence of (*E*)-caryophyllene. Morais et al. (2019) [37] reported that *Croton* species with caryophyllene as the dominant constituent exhibited higher antioxidant activity compared to standards such as thymol and butylated hydroxytoluene (BHT). Moreover, they observed that antioxidant performance was directly related to the concentration of caryophyllene. In addition, Sytykiewicz et al. (2025) [38] demonstrated similar trends in essential oils of *Juniperus communis* and *Acorus calamus*. In that study, *J. communis*, dominated by α-pinene (22%), exhibited greater antioxidant capacity than *A. calamus,* as evidenced by DPPH and ABTS values of 85.4 ± 0.8 μg/mL and 14.2 ± 0.1 μg/mL, respectively.

Consistently with these observations, the major constituents of *C. alnifolius* essential oil, such as (*E*)-caryophyllene and α-pinene, could account for its strong antioxidant activity in the ABTS assay. A possible synergistic interaction between these compounds may enhance the overall activity, given that both have been previously reported to possess significant antioxidant potential. Finally, as highlighted by Chaves et al. (2020) [39], the apparent variability in antioxidant results further underscores the importance of selecting appropriate radical-generating systems when assessing antioxidant activity.

Regarding acetylcholinesterase (AChE) inhibition, the essential oil of *C. alnifolius* exhibited an IC_50_ value of 61.74 ± 1.02 µg/mL. According to the classification proposed by Magalhães et al. (2021) [40], AChE inhibitory activity is considered high when IC_50_ < 20 μg/mL, moderate when IC_50_ < 200 μg/mL, and low when IC_50_ < 1000 μg/mL. Based on this categorization, the essential oil of *C. alnifolius* can be classified as a moderate AChE inhibitor. The inhibition of AChE is a crucial therapeutic target in the management of neurodegenerative disorders, such as Alzheimer’s disease, due to the fundamental role of this enzyme in regulating cholinergic neurotransmission by hydrolyzing acetylcholine at neuronal synapses [41]. Therefore, the inhibitory activity demonstrated by *C. alnifolius* essential oil suggests significant pharmacological potential.

To date, no previous studies have reported the AChE inhibitory activity of *C. alnifolius* essential oil. Consequently, this research represents the first scientific evidence in this line for the species. The phytochemical analysis revealed a balanced composition of (*E*)-caryophyllene, α-pinene, and germacrene D as the major constituents.

Moreover, α-Pinene, a monoterpene, has been previously reported as a potent AChE inhibitor [42]. This finding is consistent with the results of Calva et al. (2023) [43], where an essential oil with α-pinene (22.70%) as the predominant compound exhibited moderate AChE inhibition (IC_50_ = 53.08 ± 1.13 μg/mL). Similarly, in *Cyathocalyx pruniferus*, α-pinene (24.4%) and germacrene D (20.2%) were identified as the main constituents, resulting in 75.5% inhibition compared with galantamine (85.6%) [44]. These studies highlight the central role of α-pinene in conferring anticholinesterase activity, suggesting that it may be one of the main contributors to the activity observed in the present study.

Although (*E*)-caryophyllene was also identified as a major constituent, current literature provides no direct evidence of its AChE inhibitory capacity. Nevertheless, Dahham et al. (2015) [30] reported its anticancer, antimicrobial, anti-inflammatory, and antioxidant properties. Interestingly, (*E*)-caryophyllene has been identified as the main constituent in other essential oils showing notable AChE inhibition. For instance, in *Eugenia sulcata*, (*E*)-caryophyllene (24.6%) was the predominant component and demonstrated strong inhibitory activity (IC_50_ = 4.66 ± 0.48 μg/mL), compared to physostigmine (IC_50_ = 0.59 ± 0.02 μg/mL) [45].

On the other hand, germacrene D has been associated with analgesic, anti-inflammatory, and antioxidant activities [46]. Similar to (*E*)-caryophyllene, germacrene D has been identified as a major constituent in plants showing promising AChE inhibition. For example, Morocho et al. (2025) [47] reported germacrene D (21.75%) as the major constituent in the fruits of *Zanthoxylum mantaro*, which displayed an IC_50_ of 65.46 ± 1.01 μg/mL.

Taken together, these findings suggest a possible synergistic effect among the main components of *C. alnifolius* essential oil. While α-pinene appears to play the predominant role in the observed anticholinesterase activity, the combined action of (*E*)-caryophyllene and germacrene D may enhance the overall inhibitory effect, reinforcing the pharmacological relevance of this essential oil.

The biological activities observed for the essential oil of *C. alnifolius* suggest potential applications in natural antioxidant and neuroprotective formulations. However, beyond its bioactivity, the practical and economic feasibility of the extraction process must also be considered. The essential oil in this study was obtained using a Clevenger-type hydrodistillation apparatus, which is suitable for laboratory-scale extractions but not representative of industrial production systems. The low yield (0.028%) reflects both the plant’s intrinsic characteristics and the limitations of this method, including high energy consumption, long extraction time, and low recovery efficiency. In contrast, industrial-scale techniques such as continuous steam distillation, supercritical CO_2_ extraction, and microwave-assisted hydrodistillation can provide higher yields, shorter processing times, and better preservation of thermolabile compounds, while also improving environmental sustainability [48,49]. From an economic standpoint, the viability of large-scale production would depend on optimizing biomass availability, energy efficiency, and operational costs [50]. Future studies should therefore evaluate these alternative methods to determine the most sustainable and cost-effective strategies for industrial exploitation of *C. alnifolius* essential oil.

## 4. Materials and Methods

### 4.1. Plant Material

Aerial parts of *Croton alnifolius* were collected in October 2024 during the flowering stage from Yambaca, Calvas canton, Loja province, Ecuador (4°24′40″ S, 79°37′14″ W; 1663 m a.s.l.). The plant material was transported to the Bioproducts Plant of the Universidad Técnica Particular de Loja (UTPL). Drying was carried out in an electric oven (Lassele DY-330H, Ansan City, Gyeonggi-do, Republic of Korea) at 35 °C for 48 h. The taxonomic identification was confirmed by Dr. Nixon Cumbicus at the UTPL Herbarium, where a voucher specimen was deposited under the accession number HUTPL5625. The collection was performed under the authorization issued by the Ministry of Environment of Ecuador (MAATE-ARSFC-2022-2839).

### 4.2. Extraction of Essential Oil

The essential oil was obtained by steam distillation for 4 h using a Clevenger-type apparatus. Three independent distillations were performed, each with approximately 400 g of dried plant material. Each distillation yielded approximately 110 mg of essential oil. The essential oil was collected directly from the Clevenger separator using a glass Pasteur pipette and transferred into pre-weighed 2 mL amber vials. The net mass of the essential oil was determined with high accuracy using an analytical balance Radwag AS 310/C/2 (310 g × 0.1 mg; Radwag, Radom, Poland). The obtained oil was dehydrated using 20–30 mg of anhydrous sodium sulfate, added carefully to prevent any loss of material and stored at −15 °C for further analysis. The yield was calculated on a weight/weight basis (*w*/*w*) relative to the dry plant material.

### 4.3. Qualitative and Quantitative Chemical Characterization of the Essential Oil

#### 4.3.1. Gas Chromatography–Mass Spectrometry (GC–MS) Analysis

For qualitative profiling, 10 µL of essential oil was dissolved in 990 µL of HPLC-grade cyclohexane (Sigma-Aldrich, St. Louis, MO, USA), giving a final concentration of 1% (*v*/*v*). Analyses were performed on a Thermo Scientific Trace 1310 gas chromatograph coupled to an ISQ 7000 single-quadrupole mass spectrometer (Waltham, MA, USA). Separation was achieved using a TR-5MS capillary column (30 m × 0.25 mm i.d., 0.25 µm film thickness; 5% phenyl polysilphenylene-siloxane stationary phase, Thermo Fisher Scientific (Waltham, MA, USA). Helium served as the carrier gas at a constant flow of 1.0 mL min^−1^. The injector temperature was set at 230 °C and operated in split mode (1:80). The oven program began at 50 °C (held 3 min), increased at 3 °C min to 230 °C, and was maintained for 3 min. The MS detector operated in full-scan mode (40–400 *m*/*z*) with a scan rate of 0.2 s, ion source at 230 °C, and transfer line at 250 °C.

The components of the essential oil of *C. alnifolius* were identified by comparing their mass spectra with those of reference compounds showing similar Linear Retention Indices (LRIs) reported in the literature [17]. LRIs were calculated according to the method of Van Den Dool and Kratz [51], based on the retention times of a homologous series of n-alkanes C9–C22 (ChemService, West Chester, PA, USA) analyzed under the same chromatographic conditions to ensure reliable identification. Data acquisition and processing were performed using Chromeleon XPS software, version 7.2.10 (Waltham, MA, USA), and spectral matching was carried out using the NIST 17 MS library from the internal chromatogram database. A compound was considered positively identified when the calculated linear retention index (LRI) differed by no more than ±20 units from the corresponding literature value [52].

#### 4.3.2. Gas Chromatography with Flame Ionization Detection (GC–FID) Analys

Quantitative analysis was performed using a Thermo Scientific Trace 1310 gas chromatograph equipped with a flame ionization detector (FID). The chromatographic separation employed the same TR-5MS column, temperature program, and operational parameters as described for the GC–MS analysis. The detector temperature was maintained at 250 °C, with hydrogen and air supplied at constant flow rates. Quantification was based on the integration of individual peak areas, and the results were expressed as relative percentages of the total composition without the use of correction factors.

### 4.4. Biological Activity

#### 4.4.1. Antimicrobial Activity

The antimicrobial potential of the essential oil was determined using the broth microdilution assay following the procedure of Cartuche et al. [53] with slight adjustments. Minimum inhibitory concentration (MIC) values were assessed against standard American Type Culture Collection (ATCC) (Guildford, United Kingdom) strains representing common opportunistic pathogens: *Enterococcus faecium* ATCC 27270, *Staphylococcus aureus* ATCC 25923, *Staphylococcus epidermidis* ATCC 12228, *Escherichia coli* O157:H7 ATCC 43888, *Pseudomonas aeruginosa* ATCC 10145, *Candida albicans* ATCC 10231, and *Aspergillus niger* ATCC 6275. Positive control antibiotics included ampicillin (1 mg/mL) for Gram-positive bacteria, ciprofloxacin (1 mg/mL) for Gram-negative strains, and amphotericin B (250 µg/mL) for fungi. Wells containing only broth or the maximum DMSO concentration served as negative controls.

#### 4.4.2. Radical Scavenging Assay

The antioxidant capacity of the essential oil was assessed using both DPPH and ABTS radical scavenging assays as described by Cartuche et al. [53], with modifications.

For the DPPH assay, a 0.247 mM methanolic solution of 2,2-diphenyl-1-picrylhydrazyl (DPPH) was prepared to reach an absorbance of 1.1 ± 0.01 at 515 nm. Serial dilutions of the essential oil (1.0, 0.5, and 0.25 mg/mL) were mixed with the DPPH working solution (270 µL + 30 µL sample) in 96-well plates and incubated for 60 min at room temperature in the dark. Absorbance was read at 515 nm using an EPOCH 2 microplate reader (BioTek, Winooski, VT, USA). Trolox and methanol served as positive and blank controls, respectively. Results were expressed as SC_50_ values (concentration required to reduce 50% of radicals).

For the ABTS assay, ABTS radicals were generated by mixing 7.4 mM ABTS with 2.6 mM potassium persulfate and allowing the reaction to proceed for 16 h in darkness. The resulting solution was diluted with methanol to an absorbance of 1.15–1.20 at 734 nm. Serial dilutions of essential oil (1.0, 0.5, and 0.25 mg/mL) were reacted with the ABTS working solution (270 µL + 30 µL sample) for 60 min under the same conditions. Trolox served as reference antioxidant, and results were also expressed as SC_50_ values. All experiments were performed in triplicate.

#### 4.4.3. Acetylcholinesterase Inhibitory Activity

Inhibition of acetylcholinesterase (AChE) was evaluated using a colorimetric microplate assay adapted from methods [53,54]. Serial dilutions of essential oil (10–1000 µg/mL) were prepared in methanol. The reaction mixture contained phosphate-buffered saline (PBS), 5,5′-dithio-bis-(2-nitrobenzoic acid (DTNB), and acetylthiocholine iodide (ATCh) as substrate. After pre-incubation for 3 min at 37 °C, the enzyme solution was added, and the mixture was incubated for 1 h in darkness. Absorbance was measured at 412 nm using an EPOCH 2 microplate reader (BioTek). Methanol was used as the negative control, and donepezil hydrochloride served as the positive reference. IC_50_ values were obtained from dose–response curves.

## 5. Conclusions

This study was focused on obtaining and characterizing the essential oil of *Croton alnifolius*, evaluating its antimicrobial activity, and analyzing its antioxidant and acetylcholinesterase-inhibiting properties. The essential oil exhibited a complex chemical composition dominated by sesquiterpenes and hydrocarbon monoterpenes, with (*E*)-caryophyllene, α-pinene, myrcene, and germacrene D standing out as the main constituents; these were identified by using gas chromatography coupled with mass spectrometry (GC–MS and gas chromatography with a flame ionization detector (GC–FID) for quantification. To our knowledge, this study is the first report on the composition of *C. alnifolius* essential oil. Biologically, the oil showed moderate antimicrobial activity against *Enterococcus faecium* but did not exhibit a significant effect on other microorganisms analyzed. This could be due to the concentration of bioactive compounds or possible antagonistic interactions. The oil demonstrated strong antioxidant activity in the ABTS assay, but weak activity in the DPPH assay, likely due to differences in assay kinetics and the possible synergistic action of its main components. Furthermore, it showed moderate acetylcholinesterase inhibitory activity. These results highlight the potential pharmacological properties of *C. alnifolius* essential oil from natural sources, particularly its antioxidant and neuroprotective activity, which warrants further investigation. This knowledge also provides important phytochemical information about the Euphorbiaceae family and the *Croton* genus in southern Ecuador.

## Figures and Tables

**Table 1:** Chemical composition of *C. alniflius* essential oil from Ecuador.

N°	Compound	LRI ^a^	LRI ^b^	% ^c^ ± SD	MF
1	Tricyclene	922	921	0.06 ± 0.0004	C_10_H_16_
2	α-Thujene	926	924	0.59 ± 0.0081	C_10_H_16_
3	α-Pinene	933	932	14.53 ± 0.1987	C_10_H_16_
4	Camphene	950	946	0.44 ± 0.0409	C_10_H_16_
5	Sabinene	974	969	0.34 ± 0.0092	C_10_H_16_
6	β-Pinene	979	974	1.47 ± 0.0493	C_10_H_16_
7	Myrcene	992	988	9.51 ± 0.2775	C_10_H_16_
8	α-Phellandrene	1010	1002	1.73 ± 0.0435	C_10_H_16_
9	α-Terpinene	1019	1014	0.08 ± 0.0007	C_10_H_16_
10	o-Cymene	1029	1022	0.90 ± 0.0505	C_10_H_14_
11	Limonene	1032	1024	0.88 ± 0.0192	C_10_H_16_
12	β-Phellandrene	1034	1025	0.62 ± 0.0194	C_10_H_16_
13	(*E*)-β-Ocimene	1049	1044	0.53 ± 0.0242	C_10_H_16_
14	γ-Terpinene	1061	1054	0.51 ± 0.0192	C_10_H_16_
15	Linalool	1107	1105 ^d^	1.35 ± 0.0641	C_10_H_18_O
16	Safranal	1208	1197	0.24 ± 0.0108	C_10_H_14_O
17	Isobornyl formate	1236	1235	0.27 ± 0.0120	C_11_H_18_O_2_
18	Bornyl acetate	1291	1284	0.09 ± 0.0033	C_12_H_20_O_2_
19	Isoledene	1370	1374	1.70 ± 0.0380	C_15_H_24_
20	α-Ylangene	1377	1373	2.57 ± 0.0090	C_15_H_24_
21	β-Bourbonene	1385	1387	0.28 ± 0.0212	C_15_H_24_
22	β-Cubebene	1390	1387	0.32 ± 0.0261	C_15_H_24_
23	β-Elemene	1392	1389	1.06 ± 0.0972	C_15_H_24_
24	Methyl eugenol	1416	1403	0.19 ± 0.0310	C_11_H_14_O_2_
25	(*E*)-Caryophyllene	1424	1417	17.42 ± 0.4651	C_15_H_24_
26	β-Copaene	1433	1430	1.22 ± 0.0001	C_12_H_18_O_3_
27	Spirolepechinene	1452	1449	0.88 ± 0.0060	C_15_H_24_
28	β-Chamigrene	1458	1476	5.48 ± 0.1380	C_15_H_24_
29	(*E*)-Methyl isoeugenol	1472	1492	0.66 ± 0.0097	C_11_H_14_O_2_
30	*trans*-Muurola-4(14),5-diene	1480	1493	2.12 ± 0.0200	C_15_H_24_
31	Germacrene D	1486	1480	9.92 ± 0.3206	C_15_H_24_
32	Valencene	1497	1496	0.39 ± 0.0154	C_15_H_24_
33	Bicyclogermacrene	1501	1500	2.64 ± 0.0502	C_15_H_24_
34	α-Muurolene	1504	1500	1.46 ± 0.0306	C_15_H_24_
35	Premnaspirodiene	1508	1506	0.40 ± 0.0188	C_15_H_24_
36	β-Curcumene	1514	1514	1.35 ± 0.0279	C_15_H_24_
37	δ-Cadinene	1524	1522	2.65 ± 0.1419	C_15_H_24_
38	α-Cadinene	1544	1537	0.18 ± 0.0062	C_15_H_24_
39	α-Copaen-11-ol	1551	1539	0.38 ± 0.1234	C_15_H_24_O
40	Germacrene B	1566	1559	2.30 ± 0.0352	C_15_H_24_
41	Helifolen-12-al	1576	1592	0.34 ± 0.0098	C_15_H_22_O
42	Caryophyllene oxide	1593	1582	2.79 ± 0.0807	C_15_H_24_O
44	Cubenol	1638	1645	0.42 ± 0.0212	C_15_H_26_O
45	epi-α-Cadinol	1655	1638	0.71 ± 0.0449	C_15_H_26_O
46	α-Cadinol	1669	1652	0.35 ± 0.2591	C_15_H_26_O
47	n-Heptadecane	1697	1700	0.15 ± 0.0260	C_17_H_36_
49	(5*E*,9*E*)-Farnesyl acetone	1919	1913	0.17 ± 0.0004	C_18_H_30_O
	Hydrocarbon monoterpenes			32.20	
	Oxygenated monoterpenes			1.59	
	Hydrocarbon sesquiterpenes			53.11	
	Oxygenated sesquiterpenes			5.00	
	Others			2.76	
	**Total**			**94.65**	

LRI ^a^, linear retention index calculated; LRI ^b^, linear retention index from reference [17]; % ^c^, %: mean percentage content in the EO over 3 determinations; ^d^, LRI ^b^ value obtained from the NIST 17 mass spectral library [18]; SD, standard deviation; MF: molecular formula.

**Table 2:** In vitro antimicrobial activity of *C. alnifolius* essential oil expressed as minimum inhibitory concentrations (MICs).

Microorganisms	*C. alnifolius* Essential Oil (µg/mL)	Antimicrobial Agent (Positive Control)
Gram positive bacteria		Ampicilin (1 mg/mL)
*Enterococcus faecium* ATCC^®^ 27270	1000	<0.3906
*Staphylococcus aureus* ATCC^®^ 25923	>1000 µg/mL	<0.3906
*Staphylococcus epidermidis* ATCC^®^ 12228	>1000 µg/mL	<0.3906
Gram negative bacteria		Ciprofloxacin (1 mg/mL)
*Escherichia coli* (O157:H7) ATCC^®^ 43888	>1000 µg/mL	1.5625
*Pseudomonas aeruginosa* ATCC^®^ 10145	>1000 µg/mL	<0.3906
Yeasts and sporulated fungi		Amphotericin B (250 µg/mL)
*Aspergillusniger* ATCC^®^ 6275	4000	<0.098
*Candida albicans* ATCC^®^ 10231	>4000 µg/mL	<0.098

**Table 3:** Antioxidant activity of essential oil from the leaves of *C. alnifolius*.

	DPPH	ABTS	TEAC
	SC_50_ ± SD (µg/mL—µM *)		µM Trolox/g EO
*C. alnifolius*	1903.29 ± 1.26	28.43 ± 1.0	14.58 ± 71.47
Trolox (μM)	35.54 ± 1.04	29.09 ± 1.05	

* Trolox was used as a positive reference, and its values are given in µM.

## Data Availability

All data generated or analyzed during this study are included within this article.

## References

[1] Patwardhan B., Vaidya A.D.B. (2010). Natural products drug discovery: Accelerating the clinical candidate development using reverse pharmacology approaches. Indian J. Exp. Biol..

[2] Chaachouay N., Zidane L. (2024). Plant-Derived Natural Products: A Source for Drug Discovery and Development. Drugs Drug Candidates.

[3] Tongnuanchan P., Benjakul S. (2014). Essential Oils: Extraction, Bioactivities, and Their Uses for Food Preservation. J. Food Sci..

[4] Ramsey T., Shropshire C., Nagy T.R., Chambers K.D., Li Y., Korach K.S. (2020). Essential Oils and Health. Yale J. Biol. Med..

[5] Da Costa L.S., Ferreira O.O., Lobato L.G.N., De Santana Botelho A., Mali S.N., Kumar R., De Jesus Pereira Franco C., Rosa U.A., Correia Z.A., Da Silva M.P. (2024). Exploring phytochemistry, antioxidant capacity, and biological potential of essential oils obtained from Euphorbiaceae species. Phytochem. Rev..

[6] Luu-Dam N.A., Le C.V.C., Satyal P., Le T.M.H., Bui V.H., Vo V.H., Ngo G.H., Bui T.C., Nguyen H.H., Setzer W.N. (2023). Chemistry and Bioactivity of *Croton* Essential Oils: Literature Survey and *Croton hirtus* from Vietnam. Molecules.

[7] Inostroza L., Hernandez E., Casanova H., Castro A. (2011). Evaluación de la actividad leishmanicida y toxicidad aguda del extracto hidroalcohólico de los tallos de *Croton alnifolius*. Cienc. Investig..

[8] Barrera C.A., Gómez D.C., Castiblanco F.A. (2016). Importancia medicinal del género *Croton* (Euphorbiaceae). Rev. Cuba. Plantas Med..

[9] Coy-Barrera C.A., Galvis L., Rueda M.J., Torres-Cortés S.A. (2025). The *Croton* genera (Euphorbiaceae) and its richness in chemical constituents with potential range of applications. Phytomedicine Plus.

[10] Chen X., Shang S., Yan F., Jiang H., Zhao G., Tian S., Chen R., Chen D., Dang Y. (2023). Antioxidant Activities of Essential Oils and Their Major Components in Scavenging Free Radicals, Inhibiting Lipid Oxidation and Reducing Cellular Oxidative Stress. Molecules.

[11] Reddy B.H.V., Hussain S.M.S., Hussain M.S., Kumar R.N., Gupta J. (2025). Essential oils in cosmetics: Antioxidant properties and advancements through nanoformulations. Pharmacol. Res. Nat. Prod..

[12] Caro-Gamboa L.J., Forero-Castro M., Dallos-Báez A.E. (2020). Inhibición de la colinesterasa como biomarcador para la vigilancia de población ocupacionalmente expuesta a plaguicidas organofosforados. Cienc. Tecnol. Agropecu..

[13] Dobetsberger C., Buchbauer G. (2011). Actions of essential oils on the central nervous system: An updated review. Flavour Fragr. J..

[14] Hung N.H., Quan P.M., Satyal P., Dai D.N., Hoa V.V., Huy N.G., Giang L.D., Ha N.T., Huong L.T., Hien V.T. (2022). Acetylcholinesterase Inhibitory Activities of Essential Oils from Vietnamese Traditional Medicinal Plants. Molecules.

[15] Kew Science *Croton alnifolius* Lam. Plants of the World Online. https://powo.science.kew.org/taxon/urn:lsid:ipni.org:names:342011-1.

[16] Missouri Botanical Garden. *Croton alnifolius* Lam. Tropicos. https://tropicos.org/name/12800801.

[17] Adams R.P. (2007). Identification of Essential Oil Components by Gas Chromatography/Mass Spectrometry.

[18] Sabulal B., Dan M., John J.A., Kurup R., Chandrika S.P., George V. (2007). Phenylbutanoid-rich rhizome oil of *Zingiber neesanum* from Western Ghats, southern India. Flavour Fragr. J..

[19] Ellman G.L., Courtney K.D., Andres V., Featherstone R.M. (1961). A new and rapid colorimetric determination of acetylcholinesterase activity. Biochem. Pharmacol..

[20] Molares S., González S.B., Ladio A., Castro M.A. (2009). Etnobotánica, anatomía y caracterización físico-química del aceite esencial de *Baccharis obovata* Hook. et Arn. (Asteraceae: Astereae). Acta Bot. Bras..

[21] Cucho J., Mendoza-Beingolea S., Fuerte-Ruitón C., Salazar-Salvatierra M., Herrera-Calderón O. (2021). Chemical Profile of the Volatile Constituents and Antimicrobial Activity of the Essential Oils from *Croton adipatus*, *Croton thurifer*, and *Croton collinus*. Antibiotics.

[22] Valarezo E., Gaona-Granda G., Morocho V., Cartuche L., Calva J., Meneses M.A. (2021). Chemical Constituents of the Essential Oil from Ecuadorian Endemic Species *Croton ferrugineus* and Its Antimicrobial, Antioxidant and α-Glucosidase Inhibitory Activity. Molecules.

[23] Ruphin F.P., Baholy R., Sylver S., Oscar R.A., Mahamoud A., Raymond F.F., Marcelin S., Rakotoniriana H.J., Amélie R., Ngbolua K.-t.-N. (2016). GC-FID and GC/MS analyses and Antimicrobial activity of *Croton greveanus*, *C. borarium* and *C. geayi* (Euphorbiaceae) essential oils from Madagascar. J. Pharmacogn. Phytochem..

[24] Ogundajo A.L., Ogunwande I.A., Gbadamosi H.G., Giwa R., Flamini G. (2014). Chemical Composition of the Leaf Essential Oils of *Croton zambesicus* Müll.-Arg. Grown in Lagos, South-West Nigeria. Eur. J. Med. Plants.

[25] Fernández-Rosillo F., Aguirre-Vargas E.B. (2024). Variación estacional del rendimiento de extracción y actividad biológica in vitro del aceite esencial de *Lippia alba* (pampa orégano). Dekamu Agropec.

[26] Pardo F.T., Lujan A.I., Nauto N.G., Laqui-Estaña J., Quispe D.C. (2021). La presión de extracción afecta el rendimiento, las características físico-químicas y los compuestos químicos del aceite esencial de Punamuña (*Satureja bolivariana*). Int. J. Curr. Res..

[27] Çoban F., Özer H., Cakmakcı R. (2024). Altitude-Dependent Variation in Chemical Composition of Essential Oil of Origanum acutidens (Hand-Mazz.) Ietswaart. J. Agric. Prod..

[28] Maldonado F.M., Jaramillo J.V., Gilardoni G. (2021). Composición química del aceite esencial de la especie ecuatoriana *Croton rivinifolius* kunth (Euphorbiaceae). Axioma.

[29] Freires I.A., Denny C., Benso B., Alencar S.M., Rosalen P.L. (2015). Antibacterial Activity of Essential Oils and Their Isolated Constituents against Cariogenic Bacteria: A Systematic Review. Molecules.

[30] Dahham S.S., Tabana Y.M., Iqbal M.A., Ahamed M.B., Ezzat M.O., Majid A.S., Majid A.M. (2015). The Anticancer, Antioxidant and Antimicrobial Properties of the Sesquiterpene β-Caryophyllene from the Essential Oil of *Aquilaria crassna*. Molecules.

[31] Vaou N., Stavropoulou E., Voidarou C., Tsakris Z., Rozos G., Tsigalou C., Bezirtzoglou E. (2022). Interactions between Medical Plant-Derived Bioactive Compounds: Focus on Antimicrobial Combination Effects. Antibiotics.

[32] Bin Nabi H., Ahmed M., Mia S., Islam S., Zzaman W. (2025). Essential oils: Advances in extraction techniques, chemical composition, bioactivities, and emerging applications. Food Chem. Adv..

[33] Bassolé I.H., Juliani H.R. (2012). Essential Oils in Combination and Their Antimicrobial Properties. Molecules.

[34] Kusmardiyani S., Alfianti F., Fidrianny I. (2016). Antioxidant profile and phytochemical content of three kinds of *Lemon grass* grown in west Java-Indonesia. Asian J. Pharm. Clin. Res..

[35] Kuskoski E.M., Asuero A.G., Troncoso A.M., Mancini-Filho J., Fett R. (2005). Aplicação de diversos métodos químicos para determinar atividade antioxidante em polpa de frutas. Food Sci. Technol..

[36] Floegel A., Kim D., Chung S., Koo S., Chun O. (2011). Comparison of ABTS/DPPH assays to measure antioxidant capacity in popular antioxidant-rich US foods. J. Food Compos. Anal..

[37] Morais S.M., Cossolosso D.S., Silva A.A., Filho M.O., Teixeira M.J., Campello C.C., Bonilla O.H., Paula Júnior V.F., Vila-Nova N.S. (2019). Essential Oils from *Croton* Species: Chemical Composition, in vitro and in silico. J. Braz. Chem. Soc..

[38] Sytykiewicz H., Łukasik I., Goławska S. (2025). Chemical Composition, Anti-Tyrosinase and Antioxidant Potential of Essential Oils from *Acorus calamus* (L.) and *Juniperus communis* (L.). Molecules.

[39] Chaves N., Santiago A., Alías J.C. (2020). Quantification of the Antioxidant Activity of Plant Extracts: Analysis of Sensitivity and Hierarchization Based on the Method Used. Antioxidants.

[40] Magalhães V., Rios R.M., Silva F.G.C.P., Dias A.C.P. (2021). Comparative Study on the Inhibition of Acetylcholinesterase Activity by *Hyptis marrubioides*, *Hyptis pectinata*, and *Hyptis suaveolens* Methanolic Extracts. Proceedings.

[41] Saud A., Krishnaraju V., Taha A., Kalpana K., Malarkodi V., Durgaramani S., Prabhu V.V., Saleh F.A., Ezhilarasan S. (2024). Potential acetylcholinesterase inhibitors to treat Alzheimer’s disease. Eur. Rev. Med. Pharmacol. Sci..

[42] Miyazawa M., Yamafuji C. (2005). Inhibition of Acetylcholinesterase Activity by Bicyclic Monoterpenoids. J. Agric. Food Chem..

[43] Calva J., Silva M., Morocho V. (2023). Composition and Anti-Acetylcholinesterase Properties of the Essential Oil of the Ecuadorian Endemic Species *Eugenia valvata* McVaugh. Molecules.

[44] Salah P.M., Khamis S., HelmiNadri M., Kassim H., Tawang A. (2020). Chemical composition and acetylcholinesterase inhibition of the essential oil of *Cyathocalyx pruniferus* (Maingay ex Hook.f. & Thomson) J.Sinclair. Nat. Volatiles Essent. Oils.

[45] Lima B., Tietbohl L., Fernandes C., Cruz R., Botas G., Santos M., Silva-Filho M.V., Rocha L. (2012). Chemical composition of essential oils and anticholinesterasic activity of *Eugenia sulcata* Spring ex Mart. Lat. Am. J. Pharm..

[46] Ascari J., de Oliveira M.S., Nunes D.S., Granato D., Scharf D.R., Simionatto E., Otuki M., Soley B., Heiden G. (2019). Chemical composition, antioxidant and anti-Inflammatory activities of the essential oils from male and female specimens of *Baccharis punctulata* (Asteraceae). J. Ethnopharmacol..

[47] Morocho V., Eras O., Rojas T., Jiménez B., Roa M.F., Cartuche L. (2025). Biological Activity and Chemical Composition of Essential Oil from Leaves and Fruits of *Zanthoxylum mantaro* (J.F.Macbr.) J.F.Macbr. Antibiotics.

[48] Crampon C., Boutin O., Badens E. (2011). Supercritical Carbon Dioxide Extraction of Molecules of Interest from Microalgae and Seaweeds. Ind. Eng. Chem. Res..

[49] Moradi S., Fazlali A., Hamedi H.R. (2018). Microwave-Assisted Hydro-Distillation of Essential Oil from Rosemary: Comparison with Traditional Distillation. Avicenna J. Med. Biotechnol..

[50] Ahmed B., Avinash B., Nain Singh G., Graham N.T., Bohre A., Evans M., Vijay V. (2025). Biomass to bio-energy supply chain: Economic viability, case studies, challenges and policy implications in India. Sustain. Energy Technol. Assess..

[51] Van Den Dool H., Kratz P.D. (1963). A Generalization of the Retention Index System Including Linear Temperature Programmed Gas-Liquid Partition Chromatography. J. Chromatogr..

[52] Figueroa J.G., Vargas L.F. (2016). Evaluation of Sde, Sfe and Spme/Cg-Ms for Extraction and Determination of Aroma Compounds from Vilcabamba-Ecuadorian Roasted Coffee. Quim. Nova.

[53] Cartuche L., Calva J., Valarezo E., Chuchuca N., Morocho V. (2022). Chemical and Biological Activity Profiling of *Hedyosmum strigosum* Todzia Essential Oil, an Aromatic Native Shrub from Southern Ecuador. Plants.

[54] Rhee I.K., van de Meent M., Ingkaninan K., Verpoorte R. (2001). Screening for acetylcholinesterase inhibitors from Amaryllidaceae using silica gel thin-layer chromatography in combination with bioactivity staining. J. Chromatogr. A.

